# Water, sanitation, and hygiene for control of trachoma in Ethiopia (WUHA): a two-arm, parallel-group, cluster-randomised trial

**DOI:** 10.1016/S2214-109X(21)00409-5

**Published:** 2022-01

**Authors:** Solomon Aragie, Dionna M Wittberg, Wondyifraw Tadesse, Adane Dagnew, Dagnachew Hailu, Ambahun Chernet, Jason S Melo, Kristen Aiemjoy, Mahteme Haile, Taye Zeru, Zerihun Tadesse, Sarah Gwyn, Diana L Martin, Benjamin F Arnold, Matthew C Freeman, Scott D Nash, E Kelly Callahan, Travis C Porco, Thomas M Lietman, Jeremy D Keenan

**Affiliations:** The Carter Center Ethiopia, Addis Ababa, Ethiopia (S Aragie PhD, A Dagnew BSc, D Hailu BSc, A Chernet MSc, Z Tadesse MD); Francis I Proctor Foundation (D M Wittberg MPH, J S Melo MPH, K Aiemjoy PhD, B F Arnold PhD, Prof T C Porco PhD, Prof T M Lietman MD, Prof J D Keenan MD), Department of Epidemiology and Biostatistics (K Aiemjoy, Prof T C Porco, Prof T M Lietman), Department of Ophthalmology (B F Arnold, Prof T C Porco, Prof T M Lietman, Prof J D Keenan), and Institute for Global Health (Prof T M Lietman), University of California, San Francisco, CA, USA; Catholic Relief Services, Addis Ababa, Ethiopia (W Tadesse MPH); Amhara Public Health Institute, Bahir Dar, Ethiopia (M Haile PhD, T Zeru MPH); Division of Parasitic Diseases and Malaria, Centers for Disease Control and Prevention, Atlanta, GA, USA (S Gwyn MSc, D L Martin PhD); Rollins School of Public Health, Emory University, Atlanta, GA, USA (Prof M C Freeman PhD); The Carter Center, Atlanta, GA, USA (S D Nash PhD, E K Callahan MPH)

## Abstract

**Background:**

WHO promotes the SAFE strategy for the elimination of trachoma as a public health programme, which promotes surgery for trichiasis (ie, the S component), antibiotics to clear the ocular strains of chlamydia that cause trachoma (the A component), facial cleanliness to prevent transmission of secretions (the F component), and environmental improvements to provide water for washing and sanitation facilities (the E component). However, little evidence is available from randomised trials to support the efficacy of interventions targeting the F and E components of the strategy. We aimed to determine whether an integrated water, sanitation, and hygiene (WASH) intervention prevents the transmission of trachoma.

**Methods:**

The WASH Upgrades for Health in Amhara (WUHA) was a two-arm, parallel-group, cluster-randomised trial in 40 rural communities in Wag Hemra Zone (Amhara Region, Ethiopia) that had been treated with 7 years of annual mass azithromycin distributions. The randomisation unit was the school catchment area. All households within a 1·5 km radius of a potential water point within the catchment area (as determined by the investigators) were eligible for inclusion. Clusters were randomly assigned (at a 1:1 ratio) to receive a WASH intervention either immediately (intervention) or delayed until the conclusion of the trial (control), in the absence of concurrent antibiotic distributions. Given the nature of the intervention, participants and field workers could not be masked, but laboratory personnel were masked to treatment allocation. The WASH intervention consisted of both hygiene infrastructure improvements (namely, construction of a community water point) and hygiene promotion by government, school, and community leaders, which were implemented at the household, school, and community levels. Hygiene promotion focused on two simple messages: to use soap and water to wash your or your child’s face, and to always use a latrine for defecation. The primary outcome was the cluster-level prevalence of ocular chlamydia, measured annually using conjunctival swabs in a random sample of children aged 0–5 years from each cluster at 12, 24, and 36 month timepoints. Analyses were done in an intention-to-treat manner. This trial is ongoing and is registered at ClinicalTrials.gov, NCT02754583.

**Findings:**

Between Nov 9, 2015, and March 5, 2019, 40 of 44 clusters assessed for eligibility were enrolled and randomly allocated to the trial groups (20 clusters each, with 7636 people from 1751 households in the intervention group and 9821 people from 2211 households in the control group at baseline). At baseline, ocular chlamydia prevalence among children aged 0–5 years was 11% (95% CI 6 to 16) in the WASH group and 11% (5 to 18) in the control group. At month 36, ocular chlamydia prevalence had increased in both groups, to 32% (24 to 41) in the WASH group and 31% (21 to 41) in the control group (risk difference across three annual monitoring visits, after adjustment for prevalence at baseline: 3·7 percentage points; 95% CI −4·9 to 12·4; p=0·40). No adverse events were reported in either group.

**Interpretation:**

An integrated WASH intervention addressing the F and E components of the SAFE strategy did not prevent an increase in prevalence of ocular chlamydia following cessation of antibiotics in an area with hyperendemic trachoma. The impact of WASH in the presence of annual mass azithromycin distributions is currently being studied in a follow-up trial of the 40 study clusters. Continued antibiotic distributions will probably be important in areas with persistent trachoma.

**Funding:**

National Institutes of Health—National Eye Institute.

## Introduction

The SAFE strategy^1^ is the cornerstone of WHO’s plan to eliminate trachoma as a public health problem. The strategy, adopted in 1996, promotes surgery for trichiasis (ie, the S component), antibiotics to clear the ocular strains of chlamydia that cause trachoma (the A component), facial cleanliness to prevent transmission of infectious ocular and nasal secretions (the F component), and environmental improvements to provide water for washing and sanitation facilities (the E component; for reducing populations of the face-seeking fly *Musca sorbens*, a mechanical vector thought to have a role in the transmission of trachoma). The facial cleanliness and environmental components of the SAFE strategy (ie, the F and E components) were mainly recommended on the basis of observational studies that showed a relationship between improved water, sanitation, and hygiene (WASH) indicators and lower prevalences of trachoma.^2–5^

The rationale behind the facial cleanliness and environmental components of the SAFE strategy is that changes in hygiene behaviours and improvements in environmental infrastructure might be able to reduce transmission of ocular chlamydia and thus be an ideal long-term strategy for trachoma control. Trachoma was eliminated in the USA and Europe without the need for mass antibiotic distributions, perhaps because of improvements in water and sanitation infrastructure and hygiene practices. However, few randomised trials have assessed the effectiveness of WASH interventions targeting these components of the SAFE strategy, and those that have been done have typically been underpowered and of short duration.^6–15^ Given the paucity of evidence, the WASH Upgrades for Health in Amhara (WUHA) trial aimed to test the hypothesis that an integrated WASH intervention would reduce ocular chlamydia infection.

## Methods

### Study design and participants

The WUHA study was a two-arm, parallel-group, cluster-randomised trial done in Wag Hemra Zone (Amhara Region, Ethiopia). Cluster randomisation was used because the intervention was community-based, and because trachoma is a transmissible disease. The study area was situated in three contiguous districts in Wag Hemra: Sekota Zuria, Sekota Ketema, and Gazgibella. The area is arid and mountainous, and the principal occupation is agricultural. Surveys done in 2012–14 found Wag Hemra to have the highest prevalence of trachoma in the Amhara Region, with a prevalence of a key WHO-recommended trachoma indicator (ie, trachomatous inflammation–follicular among children aged 1–9 years) of 59% and the lowest coverage of several WASH indicators.^16–18^ Mass azithromycin distributions had been offered to all community members as part of routine trachoma programme activities annually in May or June from 2009 to 2015 and, in 2014, a supplemental round of azithromycin was also offered in October.

**Figure 1:**
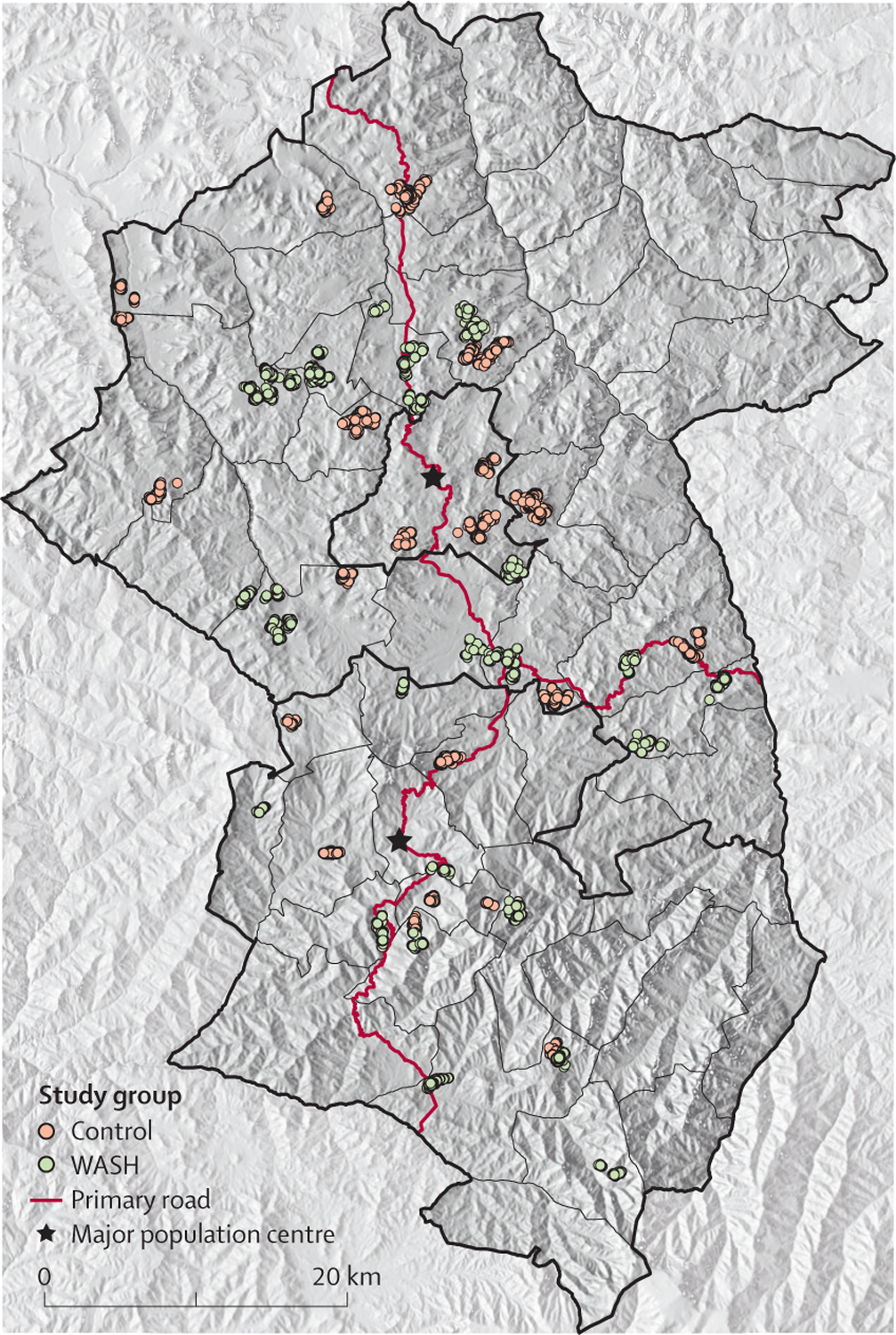
Map of study area The study area consisted of the woredas (ie, districts) in the Wag Hemra zone (Amhara Region, Ethiopia). The dots represent individual households enrolled. WASH=water, sanitation, and hygiene.

The unit of randomisation was the primary school catchment area (ie, those communities sending children to a particular school). Catholic Relief Services did a geohydrological survey before the study to identify the single most promising site for a water point (eg, hand dug well or spring development) within each catchment area. Households within a 1·5 km radius of this potential water point were eligible for community-based interventions and outcome monitoring and defined a study cluster. Only one study cluster was designated per school catchment area, creating a buffer zone between study clusters that limited contamination between the intervention and control groups (figure 1). Households in the largest population centre of each district (ie, Sekota and Asketema) were excluded since trachoma is less common in urban areas, as were households farther than a 3 h walk from the nearest place a four-wheel drive vehicle could reach. Antibiotics were not distributed in study clusters for the duration of the trial.

Ethical approval was obtained from the University of California, San Francisco; Emory University; Ethiopian Ministry of Science and Technology; and Food, Medicine, and Health Care Administration and Control Authority of Ethiopia. Verbal consent was obtained from participants or from guardians of participants due to high levels of illiteracy in the study area. The published protocol is available.^19^

### Randomisation and masking

In the trial, 20 intervention clusters were randomly assigned to an integrated WASH intervention and 20 control clusters to a WASH intervention that was delayed until the conclusion of the trial.^19^ A door-to-door enumerative census was done in all study clusters. The census was done at baseline (before randomisation) and repeated every 12 months. The name, age, and sex of all members of the study cluster households were recorded using a custom-built mobile application (Conexus, Los Gatos, CA, USA). Following completion of the baseline census and monitoring visits, the trial biostatistician randomised study clusters without stratification in a 1:1 ratio to either the WASH intervention or delayed intervention (ie, control). The randomisation sequence was generated in R (version 3). The study coordinator (SA) assigned the allocated intervention.

Allocation was concealed at the cluster-level by doing randomisation after the baseline census. Participants and field workers could not be masked due to the nature of the intervention. All laboratory staff were masked to treatment allocation, achieved by labelling all specimens with a 5-digit random number.

### Procedures

The WASH intervention consisted of both hygiene infrastructure improvements and hygiene promotion, implemented at the household, school, and community levels. Study messaging and materials were based on formative research done before the trial with government, school, and community leaders as well as community members. Hygiene promotion focused on two simple messages, repeated in different settings: first, to use soap and water to wash your or your child’s face twice per day; and second, to always use a latrine for defecation. A community water point was constructed, and all households within 1·5 km of the water point received a wash station (ie, a 25 L jerry can with faucet), a mirror, a WASH education picture book, and a monthly supply of four bars of soap. Salaried hygiene promotion workers visited households to provide hygiene education and to help household members identify specific hygiene gaps and goals. Hygiene training sessions were delivered by community leaders, priests, and government-sponsored health development army volunteers, and these individuals were asked to stress hygiene messages in their public encounters. Government policy prevented the study from directly building latrines but, during their home visits, the hygiene promotion workers encouraged households to use their own resources to build a new latrine or rehabilitate an existing one, and they promoted the use of latrines. School-based interventions included a primary school hygiene curriculum, teaching aids, and a WASH club instruction manual. Participants were instructed to report adverse events to their assigned hygiene promotion worker. Additional intervention details are described elsewhere.^19^

Both intervention and control clusters continued to receive routine health promotion services administered by the government’s Health Extension Program.^20^ This programme employs two female health extension workers for each government-run health post and requires that 75% of their time be spent in the community for health promotion. The programme includes a package of 17 essential health services in four domains: family health; disease prevention and control; hygiene and environmental sanitation; and health education and communication. The government Health Extension Program had a similar function to the intervention’s hygiene promotion workers, except that WUHA’s hygiene promotion workers had much greater frequency of contact and focused only on hygiene messages. Hygiene promotion workers in WUHA were typically responsible for only one to two study clusters and they lived in their assigned community, allowing them to devote much more time to learn about the hygiene challenges of the community and to tailor their messaging for that particular community. In contrast, government health extension workers were responsible for all communities served by the health post—approximately seven to eight communities in the study area—and hygiene promotion was only one small part of their messaging. Notably, the government programme did not provide the WASH infrastructure or school-based components of the study intervention.

Monitoring visits were done at baseline (ie, pre-randomisation) and at 12, 24, and 36 months afterwards, and occurred approximately 1 month following each census. Using the census as a sampling frame, a stratified random sample was selected at each monitoring visit, with 30 individuals per age stratum (ages 0–5 years and 6–9 years at all visits; and age ≥10 years at months 0 and 36). Separate cross-sectional random samples were drawn for each visit, so individuals selected at one visit might or might not have been selected at a subsequent visit. Those selected for monitoring were offered conjunctival swabbing of the everted right upper eyelid with a Dacron swab (Puritan Medical Products, Guildford, ME, USA). Swabs were stored on ice in the field, then at ‒20°C. DNA was extracted from each swab and then combined into pools of five, with sampling stratified by study cluster and age group. Pools were processed for the presence of *Chlamydia trachomatis* DNA with the Abbott RealTime assay (Abbott Molecular, Des Plaines, IL, USA) on the m2000 platform. The assay provides both qualitative and quantitative results, with the latter standardised against quantified elementary body suspensions provided by the University of California, San Francisco laboratory.^18^ Positive pools from the two younger age strata (0–5 years and 6–9 years) were subsequently tested individually. Cluster-level age-stratified prevalence estimates for the older age group (≥10 years) were determined from pooled results using maximum likelihood methods (except if more than 80% of pools for a cluster were positive, in which case individual swabs from positive pools were tested).^21^ Dried blood spots were collected in the 0–5 year age group on TropBio (Cellabs, Sydney, NSW, Australia) filter paper and allowed to air dry at ambient temperature while in the field, and then stored at −20°C. Dried blood spots were shipped to the Centers for Disease Control and Prevention (Atlanta, GA, USA), where they were processed for antibodies to chlamydial Pgp3 and CT694 with a multiplex bead assay on the Luminex platform (Bio-Rad Laboratories; Hercules, CA, USA) and classified as seropositive or seronegative according to previously described protocols.^22^

### Outcomes

The prespecified primary outcome was the cluster-level prevalence of ocular chlamydia infection among children aged 0–5 years across the three annual follow-up visits at 12, 24, and 36 months. The prespecified secondary outcomes that we report here were cluster-level ocular chlamydia infection among children aged 6–9 years across the three annual follow-up visits and among those aged 10 years and older at 36 months; quantitative chlamydial load among children aged 0–5 years across the three annual follow-up visits; and serological IgG response to the chlamydial antigens Pgp3 and CT694 among children aged 1–2 years at the 36-month timepoint. The age range of the serological analysis was chosen to reflect active transmission during the study period and, therefore, excluded children aged younger than 12 months due to the persistence of maternal antibodies, and excluded children aged 36 months and older since they could have been exposed to ocular chlamydia before the beginning of the study. Prespecified secondary outcomes reported elsewhere^19^ were clinical trachoma grades from conjunctival photography (in all ages at 36 months), nasopharyngeal pneumococcal macrolide resistance (in children aged 0–5 years at 36 months), anthropometric indicators (in children aged 0–5 years at 36 months), soil-transmitted helminth infection and load (in children aged 0–9 years at 12 months), and serological IgG responses to enteric pathogens (in children aged 1–2 years at 36 months).

### Statistical analysis

With 20 clusters in each group, the trial had approximately 80% power to detect an 8-percentage point difference in prevalence, assuming a two-sided alpha level of 0·05 and SD of 0·10. In the primary prespecified analysis, the prevalence of ocular chlamydia among children aged 0–5 years was compared between the two treatment groups. Cluster-level prevalence estimates from months 12, 24, and 36 were modelled in a mixed-effects linear regression model, using cluster-level baseline chlamydia prevalence, study month, and treatment assignment as covariates and a random intercept for study cluster.

A similar approach was used for the secondary chlamydia outcomes in the 6–9 years and 10 years and older age strata. For the secondary chlamydial load outcome, a cluster-level index was estimated for the 0–5 year population of each cluster at each timepoint as the mean of the log-transformed chlamydial load, including only those children with positive PCR results. This cluster-level index was analysed in a mixed-effects linear regression model similar to the primary analysis, except for being weighted by the number of positives per community. Seropositivity was defined as the presence of antibodies against both Pgp3 and CT694. Cluster-level serological outcomes at the final 36-month timepoint were compared between groups in a linear regression model adjusted for baseline seroprevalence.

All analyses were done in an intention-to-treat fashion without adjustment for post-randomisation covariates; statistical significance was determined by Monte Carlo permutation of the regression coefficient (10 000 permutations). Missing outcome data were not encountered given the study’s design (ie, repeated cross-sectional random samples). Between-community variability was expressed for the outcome measures as an intraclass correlation coefficient (ICC), estimated with a resampling method for binary outcomes (R package ICCbin) and with the ANOVA method for continuous outcomes (R package ICC). All analyses were done with R (version 4). The study was registered on ClinicalTrials.gov (NCT02754583) and overseen by a Data and Safety Monitoring Committee. No interim analyses were done.

### Role of the funding source

The funder of the study had no role in study design, data collection, data analysis, data interpretation, or writing of the report.

## Results

**Figure 2:**
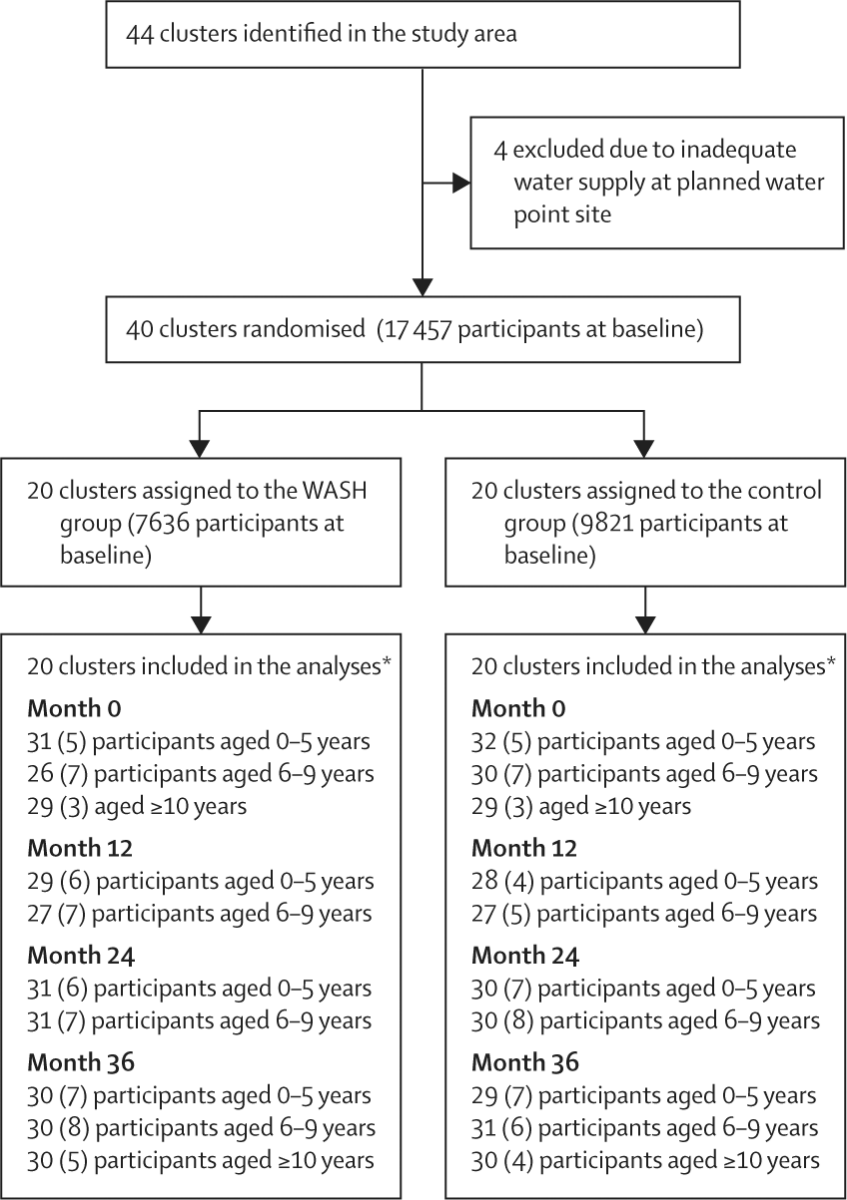
Trial profile Participant data are the mean number of individuals randomly sampled (SD) per cluster in each age stratum. WASH=water, sanitation, and hygiene. *20 clusters included in the ocular chlamydia outcomes and seroprevalence outcomes.

Between Nov 9, 2015, and March 5, 2019, 40 of 44 clusters assessed for eligibility were enrolled and randomly allocated to the trial groups and were assessed for the primary and secondary outcomes (20 clusters each); four clusters were excluded due to inadequate water supply (figure 2). The population of the 20 intervention communities was slightly lower than that of the 20 control communities, but the baseline characteristics were otherwise well balanced (table). The baseline prevalence of ocular chlamydia among children aged 0–5 years was similar in both groups, with a mean prevalence of 11% (95% CI 6–16) in the WASH group and 11% (5–18) in the control group, although the mean chlamydial load index per community was slightly higher in the WASH group (4·5, 95% CI 3·3–5·7 in the WASH group *vs* 2·3, 1·3–3·4 in the control group). The seroprevalence of chlamydia among children aged 1–2 years was similar in the two groups at baseline (20%, 95% CI 11–30 in the WASH group and 18%, 11–25 in the control group). Some between-cluster variability was observed for each of these indicators when assessed among all 40 communities at baseline, with an ICC of 0·12 (95% CI 0–0·29) for ocular chlamydial infection among children aged 0–5 years, 0·32 (0·16–0·52) for chlamydial load among children aged 0–5 years, and 0·06 (0–0·24) for seroprevalence among children aged 1–2 years. The intervention was implemented in all study communities and none of the control communities inadvertently received the intervention. No communities were lost to follow-up after randomisation, and no adverse events were reported during the study period. No study clusters refused the intervention or monitoring visits; 17 individuals in the WASH group and 26 in the control group refused a monitoring visit over the four study timepoints.

The full WASH intervention was implemented over the first year of the study. Intervention fidelity is reported separately.^23^ All communities had: a water point constructed; delivery of wash stations and WASH education picture books to at least 90% of the households; soap deliveries at least nine times per year; and a visit by the hygiene promotion worker at least six times per year to at least 90% of households. All 20 intervention schools implemented the hygiene curriculum. Adherence to the intervention was evident from annual surveys that found significantly greater use of WASH infrastructure in intervention clusters (eg, at month 36, household wash station use was observed in 51% of households in the intervention group compared with 0% in the control group, and latrine use was observed in 47% of households in the intervention group compared with 26% in the control group) and more self-reported WASH behaviours (eg, at month 36, 72% of preschool children in the intervention group had their face washed with soap the previous day compared with 40% in the control group, and 49% of adults in the intervention group used a latrine the previous day compared with 26% in the control group).

**Figure 3:**
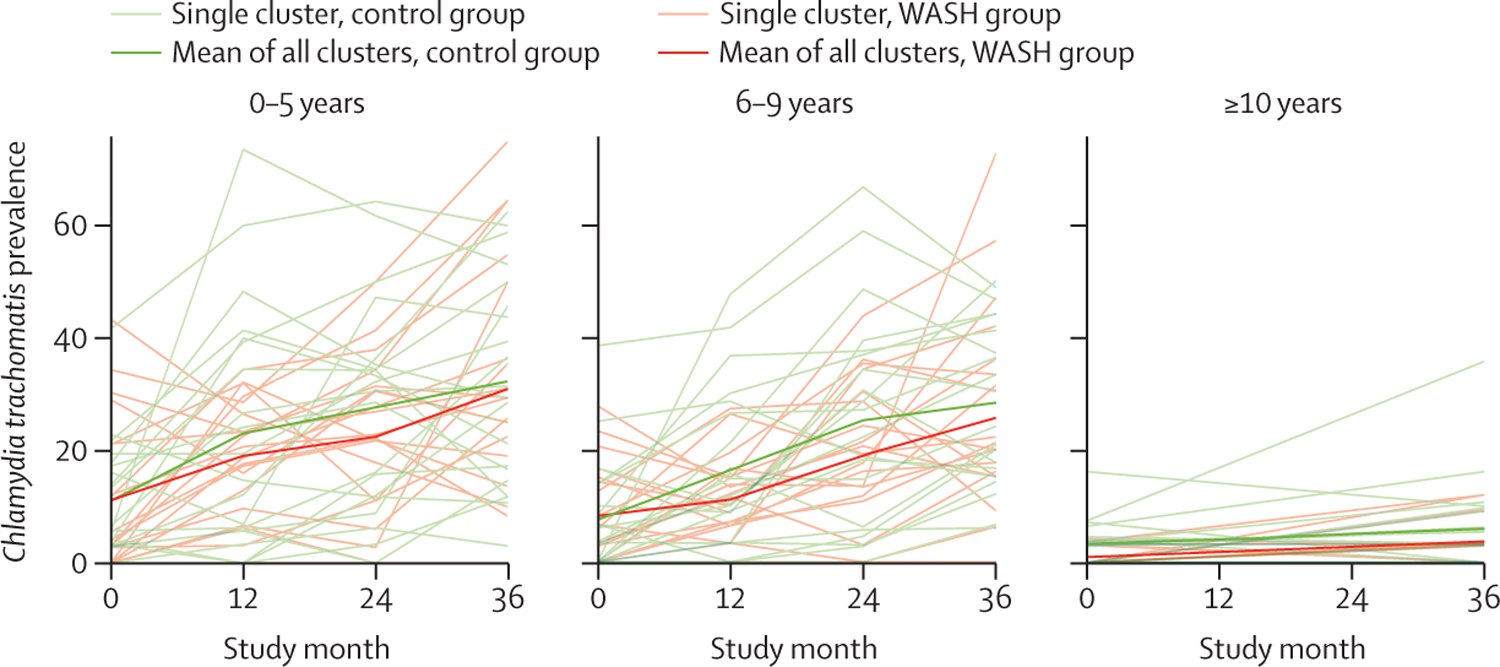
Cluster-level prevalence of ocular chlamydia over the study period The presence of *Chlamydia trachomatis* was assessed from conjunctival swabs done on an age-stratified random sample from each cluster (ages 0–5 years, 6–9 years, and ≥10 years). WASH=water, sanitation, and hygiene.

The prevalence of ocular chlamydia increased over time in all age groups and in both study groups (figure 3; appendix 2 pp 2–4). At month 36, the mean prevalence of ocular chlamydia among children aged 0–5 years was 32% (95% CI 24 to 41) in the WASH group and 31% (21 to 41) in the control group. After adjusting for prevalence at baseline (ie, the prespecified primary analysis), the prevalence of ocular chlamydia did not significantly differ between the two groups across the three post-randomisation timepoints in the WASH versus the control group (risk difference 3·7 percentage points; 95% CI –4·9 to 12·4; p=0·40). Similarly, secondary analyses of prevalence in the other age strata, the prevalence of ocular chlamydia also did not differ between the WASH and control groups in the stratum aged 6–9 years (5·0; −2·0 to 12·1; p=0·16) nor those aged 10 years (0·6; −3·6 to 4·9; p=0·78). Square root transformation of prevalence outcomes was considered but did not improve normality of model residuals.

**Figure 4:**
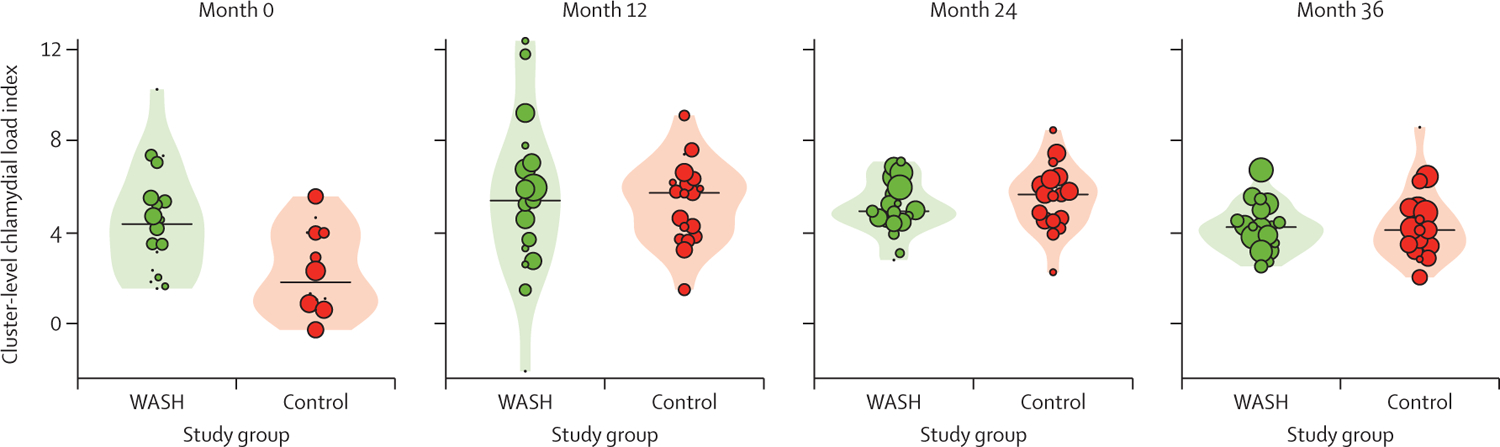
Cluster-level ocular chlamydia load among children aged 0–5 years over the study period Each dot represents a cluster, sized proportional to the number of positive chlamydia results. The range of values can be observed from the lowest and highest dot. The violin plots show the distribution and the black horizontal line represents the median of the chlamydial load index, defined as the cluster-level mean of the log-transformed estimates of chlamydial elementary bodies (ie, the infectious extracellular form of *Chlamydia trachomatis*) in each cluster. WASH=water, sanitation, and hygiene.

The mean community-level chlamydia load index among children aged 0–5 years at the month-36 visit was 4·2 (95% CI 3·7 to 4·7) in the WASH group and 4·3 (3·5 to 5·1) in the control group (figure 4; appendix 2 p 5). A repeated-measures analysis found no evidence of a difference in load between the WASH and the control groups during the trial (risk difference 0·1 units; 95% CI −0·5 to 0·7).

The seroprevalence of chlamydia among children aged 1–2 years was 33% (95% CI 21 to 45) in the WASH group versus 31% (20 to 42) in the control group at month 36, with no evidence of a significant difference (risk difference [adjusted for baseline seroprevalence] 1·3 percentage points; 95% CI −15·8 to 13·2; appendix 2 p 6).

## Discussion

The WUHA trial found no evidence of reduced ocular chlamydia infection from an integrated WASH intervention for trachoma in an area with hyperendemic trachoma following 7 years of mass azithromycin distributions. The intervention was based upon formative research and carried out with attention to implementation fidelity. The trial results suggest that, in areas with hyperendemic trachoma that have received many rounds of mass azithromycin distributions, a programme that delivers water and sanitation infrastructure and hygiene promotion, but does not include antibiotics, might not achieve trachoma elimination.

Numerous observational studies have suggested an association between various WASH indicators and trachoma.^24^ However, the causal pathway by which WASH would reduce trachoma prevalence has not been established due to a lack of data from adequately powered randomised trials of WASH interventions for trachoma. The few randomised trials of the facial cleanliness and environmental components for trachoma have been small, and typically studied single interventions, such as latrine or water point construction (appendix 2 p 7).^6–15^ Only one study mentioned that the intervention was based on formative research done specifically with the aim of developing an appropriate intervention.^8,25^ Data on intervention fidelity were either scarce or not reported at all. Previous studies were not powered to detect a small effect size, and some did not account for cluster-randomisation when doing the statistical analysis in their study. The present study addressed these limitations by providing an integrated WASH intervention with both infrastructure and behaviour-change components to a large number of clusters, which were delivered by hygiene promotion workers residing in communities and by teachers at primary schools.

Analysis of fidelity outcomes in this trial showed significant improvements in water and sanitation infrastructure and positive changes in self-reported hygiene behaviours in the WASH intervention group.^23^ And yet, the final prevalence of ocular chlamydia was not significantly different in the two treatment groups. Several reasons could explain the null result. The trial took place in a region of Ethiopia with some of the highest trachoma prevalence in all of Ethiopia and the world, despite over a decade of annual mass azithromycin distributions.^26^ Prevalence estimates for children aged 6–9 years were nearly as high as those in preschool children (aged ≥5 years), which is noteworthy since the trachoma burden is usually much greater among preschool children.^27^ The burden of infection in this study area might have been too high for hygiene interventions to have an effect, especially since antibiotics were not distributed concurrently. Additionally, the various components of the intervention might not have been intensive enough—for example, adding a single community water point could be insufficient to substantially change water collection or storage practices, and monthly hygiene promotion visits might not be adequate. However, the intervention in WUHA probably had more financial resources, addressed more elements of WASH, and was administered with greater attention to fidelity than would be possible in most trachoma programmes.

Several attempts have been made to show the positive health effects that could result from improved WASH. The SHINE and WASH Benefits trials were cluster-randomised trials set in Zimbabwe, Bangladesh, and Kenya that compared integrated WASH strategies for diarrhoea and growth.^28–30^ The results of the Bangladesh trial provided evidence that WASH interventions could reduce caregiver-reported diarrhoea; however, WASH had no effect on caregiver-reported diarrhoea in Zimbabwe or Kenya, and it had no effect on linear growth at any of the sites.^28–30^ Like WUHA, the interventions in these trials were more intensive than would be expected to be sustainable by a governmental or non-governmental health programme, but they were designed to provide proof-of-concept for the existence of a causal relationship between WASH and health outcomes. SHINE and WASH Benefits study teams concluded that, although the interventions were probably delivered with higher fidelity than might be expected from a health programme outside of research, the strength of their interventions might have ultimately been insufficient to meaningfully interrupt transmission of diarrhoea-causing pathogens in the African sites.^31^ The same challenge could apply to WUHA with respect to ocular chlamydia. Trials of more intensive interventions, with more frequent than monthly hygiene messaging or additional intervention components (eg, cleaning of shared sleeping surfaces or fly control), might be required to better understand the role of WASH for trachoma, although the feasibility and sustainability of such interventions remain unclear.

No mass azithromycin distributions were planned during implementation of WUHA. The study area had received 7 years of mass azithromycin distributions, so the burden of ocular chlamydia was expected to be very low at the onset of the trial. We hypothesised that the WUHA intervention would prevent transmission of ocular chlamydia, and purposefully did not schedule mass azithromycin distributions out of concern that antibiotic treatments would overpower any effect of the WASH intervention. However, the rate of chlamydia transmission was high in the study area, for both the strata aged 0–5 and 6–9-years. The prevalence of infection was higher than anticipated at baseline and, in the absence of antibiotics, infection increased in both the intervention and control groups throughout the study period. It is possible that the implementation of a WASH intervention alone will be unable to meaningfully interrupt transmission in such a hyperendemic setting. Given these results, we instituted annual mass azithromycin distributions to all communities following the conclusion of the trial, while maintaining the WASH intervention in the 20 communities originally randomly assigned to the WASH group. This continuation study, which will be running until April, 2023, will help determine whether implementing the A, F, and E components of the SAFE strategy are more effective than just the A component alone.

This trial had several limitations. The various aspects of the intervention were rolled out over the first year of the study and fully implemented for the final 2 years. It might have been difficult for the hygiene promotion activities to maintain momentum. Moreover, hygiene behaviour changes, although markedly greater in the WASH group, were not universal, and much of the evidence for adherence did not become apparent until the 24-month household survey.^23^ The trial was designed as an efficacy trial, to provide proof of concept that WASH could reduce transmission of trachoma. Some components of the intervention (eg, monthly soap distributions or hygiene promotion workers) might not be feasible or sustainable for some trachoma programmes. Both intervention and control communities received routine government hygiene promotion throughout the trial, which might have made the two treatment groups more similar and biased toward the null. The trial excluded remote communities due to logistical concerns, but it is possible that such remote communities would be the most marginalised of the area and perhaps the most likely to experience a benefit from a WASH intervention. Finally, the results might not be generalisable to areas with less prevalent trachoma, or to communities receiving concurrent antibiotics.

In summary, an integrated WASH intervention, implemented without adjunctive antibiotics, did not reduce transmission of ocular chlamydia over the 3-year study period when administered to Ethiopian communities with hyperendemic trachoma that had previously been treated with 7 years of annual mass azithromycin distributions. The trial is being continued with the addition of annual mass azithromycin distributions to all study communities, to determine whether the WASH intervention could be of more benefit when administered with concurrent mass antibiotics.

## Supplementary Material

1

2

## Figures and Tables

**Table:** Baseline characteristics per study cluster by study group, as assessed from a complete enumerative census

	Control group (n=20 clusters)	WASH group (n=20 clusters)
Households	110 (74–147)	87 (63–112)
Individuals	491 (326–656)	382 (273–490)
Age		
0–5 years	18% (17–20)	18% (17–20)
6–9 years	12% (12–13)	13% (12–14)
≥10 years	69% (68–71)	69% (67–70)
Sex		
Female	51% (50–52)	51% (50–52)
Male	49% (48–51)	49% (48–51%)
Distance from Sekota, km	17·6 (12·9–22·3)	22·0 (16·4–27·7)
Altitude, m	2289 (2119–2459)	2327 (2153–2501)
Household language		
Amharic	57% (35–78)	66% (45–88)
Himstagna	43% (22–65)	34% (12–55)
Other	<1% (0–1)	0
Household mobile phone	4% (2–6)	6% (1–10)


Data are mean (95% CI).

## Data Availability

Community-level data are available in appendix 2 (pp 2–6). A protocol has been published.^19^ The manual of procedures and statistical analysis plan are available.

## Research in context

### Evidence before this study

We searched PubMed from inception to Nov 29, 2021, for studies published in English, using the search terms “(water[Title] OR sanitation[Title] OR hygien*[Title] OR latrine*[Title] OR education[Title] OR wash*[Title]) AND trachoma*[Title] AND trial[Title/Abstract]”. Many observational studies have found infectious trachoma to be associated with several key hygiene indicators, including a longer distance to the primary water source, absence of a latrine, and presence of ocular and nasal discharge. Very few randomised trials have determined whether water, sanitation, and hygiene (WASH) interventions can affect trachoma control. Most of the trials that have been done have had small sample sizes and addressed a single component of hygiene; none have found a WASH intervention that is effective for interrupting transmission of the causative organism, *Chlamydia trachomatis*.

### Added value of this study

The WASH Upgrades for Health in Amhara (WUHA) study was a two-arm, parallel-group, cluster-randomised trial designed to assess the efficacy of a WASH intervention for limiting the transmission of trachoma. WUHA addressed some limitations of previous studies by implementing an integrated WASH intervention, based on formative research. Infrastructure and behavioural intervention components were delivered at the school, community, and household levels. 40 communities were enrolled in an area with hyperendemic trachoma in the absence of concurrent antibiotic treatments, increasing the statistical power of the study. At the end of the 3-year study period, ocular chlamydia increased in both the WASH and control groups, and no difference was detected between these groups.

### Implications of all the available evidence

The scope, size, and rigour of this trial provide more definitive evidence suggesting the lack of a strong effect of a WASH intervention, when used independently of other interventions, for preventing transmission of trachoma. The trial might not be generalisable to settings with less prevalent trachoma or with ongoing mass azithromycin distributions.
