# Supplementary material for: Water, sanitation, and hygiene for control of trachoma in Ethiopia (WUHA): a two-arm, parallel-group, cluster-randomised trial
Source: Lancet Glob Health. Author manuscript; Available in PMC 2022 Aug 9. (PMC9360557; doi:10.1016/S2214-109X(21)00409-5)
Supplement: 1 [file NIHMS1827832-supplement-1.pdf]

THE LANCET
Global Health

Supplementary appendix 1

This translation in Amharic was submitted by the authors and we reproduce it as supplied. It has not been peer reviewed. *The Lancet*'s editorial processes have only been applied to the original in English, which should serve as reference for this manuscript.

ይህ የአማርኛ ትርጉም ተመራማሪዎቹ ባዘጋጁት መሰረት የቀረበ እና በሌላ ወገን ያልተገመገመ መሆኑን እናስገነዝባለን። በላንሴት የአርትዖት ሂደቶች የተገመገመው በእንግሊዝኛ ቋንቋ የቀረበው ጽሑፍ ብቻ በመሆኑ ለዚህ ጽሑፍ ማጣቀሻ ሆኖ ማገልገል ያለበት በእንግሊዝኛ የተዘጋጀ መሆኑን እናሳስባለን።

Supplement to: Aragie S, Wittberg DM, Tadesse W, et al. Water, sanitation, and hygiene for control of trachoma in Ethiopia (WUHA): a two-arm, parallel-group, cluster-randomised trial. *Lancet Glob Health* 2022; **10:** e87–95.

Water, sanitation, and hygiene for control of trachoma in Ethiopia (WUHA): a two-arm, parallel-group, cluster-randomised trial

ማጠቃለያ

ዳራ፡ የአለም ጤና ድርጅት (WHO) ትራኮማን ለመቆጣጠር የ***ማመንን*** (SAFE) ሥልትን መጠቀምን ይመክራል፤ ***ማመንን*** ሲተነተን የአይን ቆብ ሽፋሽፍት ጸጉር ወደ ውስጥ መቀልበስን በአይን ቆብ ቀዶ ህክምና **ማ**ስተካከል ፣ ለትራኮማ በሽታ መንስኤ የሆነውን ተህዋስ (ክላሚዳያ ትራኮማቲስ) በማስወገድ ስርጭቱን ለመቀነስ **መ**ድሀኒት መውሠድ፣ ከአይን እና አፍንጫ በሚወጣ ፈሳሽ አማካኝነት የሚተላለፍበትን መንገድ ለመቀነስ ፊትን **ን**ጹህ ማድረግ እና የበሽታውን ሥርጭት ለመቀነስ የውሃ አቅርቦት እና መጸዳጃ ቤት በመጠቅም የአካባቢን **ን**ጽህናን ማሻሻል:: ነገር ግን ከ***ማመንን*** ሥልት ውስጥ ፊትን **ን**ጹህ ማድረግ እና የአካባቢን **ን**ጽህና መጠበቅ ተግባራትን ማከናወን የበሽታውን ሥርጭት ለመቀነስ ያለውን ውጤታማነት የሚያረጋግጥ በሳይንሳዊ የሙከራ ምርምር (randomised trials) የተደገፈ በቂ ማስረጃ እስካሁን የለም፡፡ የዚህ ጥናት አላማ የተቀናጀ የውሃ አቅርቦት የግል እና የአካባቢ ንፅህና አጠባበቅ (WASH) ተግባራትን ማከናወን የትራኮማ ሥርጭትን በመቀነስ ረገድ ውጤታማነቱን ማረጋገጥ ነው፡፡

ዘዴ: ይህ ጥናት የተካሄደው በዋግህምራ ብሄረሠብ ዞን (በአማራ ብ/ክ/መ, ኢትዮጵያ) 40 በሚሆኑ የገጠር ነዋሪዎች ወይም ማህበረሠቦች ሲሆን ለጥናቱ የተመረጡ ጥምር መንደሮችን (ክላስተር) አቻ በሚሆኑ ሁለት ቡድኖች በነሲብ የምደባ የሙከራ ጥናት ዘዴ መሠረት ለሁለት ከፍሎ በማዋቀር ነው፡፡ የዋግህምራ ዞን በትራኮማ ቁጥጥር መርሃግብር መሠረት በየአመቱ ከ ሠባት ዙር በላይ የማህበረሰብ አቀፍ የትራኮማ መድሃኒት እደላ (MDA) ተጠቃሚ የሆነ ዞን ነው፡፡ በትምህርት ቤት ዙሪያ የሚገኙ (ለትምህርት ቤቱ መጋቢ) መንደሮችን የሚያካልል ሥፍራ ለክላስተሮች የነሲብ ምደባ (randomization unit) መስፈርት ሆኖ አገልግሏል። በዚሁ የትምህርት ቤት ዙሪያ በሚገኙ መንደሮች ውስጥ የሚገኝን የውሃ ተቋም ወይም የውሃ መገኛ ሊሆን የሚችል ቦታን እንደ ማዕከል በመውሰድ በ 1.5 ኪ.ሜ. ዙሪያ ክልል ውስጥ የሚገኙ አባወራዎችን በአንድ ክላስተር ውስጥ በማካተት የጥናቱ ተሳታፊ እንዲሆኑ ተደርጓል። ሁሉም ለጥናቱ የተመረጡት ክላስተሮች የማህበረሰብ አቀፍ የትራኮማ መድሃኒት እደላ ተጠቃሚነትን በማቋረጥ እና በነሲብ የምደባ ዘዴ አማካኝነት አቻ ለአቻ (1:1 ratio) በሆነ ምደባ ከፊሎቹ የውሃ አቅርቦት ፤ የግል እና አካባቢ ንፅህና አጠባበቅ (WASH) መርሃ ግብር (ኢንተርቬንሺን) ተጠቃሚ ሲሆኑ ሌሎቹ ደግሞ ጥናቱ እስኪጠናቀቅ ድረስ ከመጠቀም በመታቀብ (ኮንትሮል) ሆነው አገልግለዋል። ከኢንተርቬንሺኑ ተፈጥሯዊ ሁኔታ የተነሳ ከላቦራቶሪ ባለሙያዎች በስተቀር የጥናቱ ተሳታፊዎችም ሆኑ የመስክ ሠራተኞችን የትኛው ክላስተር ለኢንተርቬንሺን ወይም ኮንትሮል እንደተመረጠ ማወቅ እንዳይችሉ ማድረግ አልተቻለም። የተቀናጀ የውሃ አቅርቦት፤ የግል እና የአካባቢ ንፅህና አጠባበቅ (WASH) ትግበራ የሚከተሉትን ተግባራት ያካተተ ነበር : የ WASH ተቋማትን ማሻሻል እና ግብአትን ማሟላት (ለምሳሌ; የውሃ ተቋማትን መገንባት) እንዲሁም በመንግስት ተቋማት፤ ትምህርት ቤቶች እና በማህበረሠቡ መሪዎች አማካኝነት በአባዎራ ፡ በትምህርት ቤት እና በ ማህበረሠብ ደረጃ የግል እና የአካባቢ ንፅህና ትምህርት መስጠትና እና ግንዛቤ የመፍጠር ተግባራት ናችው። የግል እና የአካባቢ ንፅህናን ማስተዋወቅ በሁለት ቀላል መልዕክቶች ላይ ያተኮረ ነበር እነርሱም ፡ የእርስዎን ፊት ሲታጠቡም ሆነ የልጅዎን ፊት ሲያጥቡ ውሃና ሳሙና ይጠቀሙ እንዲሁም ሲፀዳዱ ሁል ጊዜ መፀዳጃ ቤት ብቻ ይጠቀሙ የሚሉ ናቸው። የጥናቱ ዋና ተጠባቂ ውጤት የነበረው በማህበረሠቡ ውስጥ ያለውን ለትራኮማ መንስኤ የሆነው ተህዋስ (ኦኩላር ክላምዲያ) የሥርጭት መጠን ማወቅ ሲሆን እድሜቸው ከ 0 – 5 አመት በሆኑና በነሲብ ናሙና ለምርመራ ከተመረጡ ህፃናት ከላይኛው የአይን ቆብ ሥር በሚወሠድ የስዋብ (swab) ናሙና ምርመራ አማካኝነት

ነው። ይህ ምርመራ በሁሉም ክላስተሮች በየአመቱ ለሦሥት ተከታታይ አመታት ተከናውኗል። በዚህ ጥናት an intention-to-treat data analysis ዘዴን በመጠቀም መረጃው ተተንትኗል። ይህ ጥናት በ ClinicalTrials.gov, (NCT02754583) ተመዝግቧል።

ግኝት፡ ጥናቱ በተጀመረበት ከ ጥቅምት 29, 2008 ዓ. ም. እሥከ የካቲት 26, 2011 ዓ. ም. ውስጥ ከ ተመረጡት 44 ክላስተሮች ውስጥ መስፈርቱን ያሟሉ 40 ክላስተሮች 20 ዎቹ ለ ኢንተርቬንሺን የቀሩት 20 ዎቹ ደግሞ ለ ኮንትሮል በ ነሲብ (ራንደማይዜሺን) ዘዴ መሠረት ተመድበዋል። በጥናቱ የመጀመሪያ መነሻ ዳሠሳ ጥናት (baseline) መሠረት በ 20ዎቹ ኢንተርቬንሺን ክላስተሮች ከ 1,751 አባወራዎች 7,636 ሠዎች እንዲሁም በ 20 ዎቹ የኮንትሮል ክላስተሮች ከ 2,211 አባወራዎች 7,821 ሠዎች የጥናቱ ተሳታፊ ሆነው ተካተዋል። በጥናቱ መነሻ (baseline) ምርመራ ውጤት መሠረት እድሜቻው ከ 0 – 5 አመት በሆኑ ህፃናት የ አኩላር ክላሚዲያ ሥርጭት መጠን በኢንተርቬንሺን 11 % (95% CI 6 to 16) እንዲሁም በኮንትሮል ክላስተሮች 11 % (5 to 18) ነበር ። በ 36ኛው ወር ጥናት ምርመራ መሠረት የ አኩላር ክላምዲያ ሥርጭት መጠን በ ሁለቱም ግሩፕ የጨመረ ሲሆን 32 % (24 to 41) በኢንተርቬንሺን እና 31 % (21 to 41) በኮንትሮል ክላስተሮች ሆኖ ተገኝቷል። ለአኩላር ክላምዲያ የተጋላጭነት መጠን ልዩነት (risk difference) (የሦሥቱንም ኢንተርቬንሺን አመታት የአኩላር ክላምዲያ ሥርጭት መጠን መሠረት በማድረግ እና የጥናቱ የሥርጭት መጠን ማስተካካያ ከተደረገ በኋላ (after adjustment for prevalence at baseline) ሲሠላ 3·7 ፐርሠንት ፖይንትስ (95% CI –4·9 to 12·4; p=0·40) ሆኗል። በዚህ ጥናት ምንም አይነት የጎንዮሽ ጉዳት ከሁሉም ክላስተሮች ሪፖርት አልተደረገም።

የጥናቱ ግኝት ትርጉም፡ **የማመንን** (SAFE) ሥልት ክፍል የሆኑትን ፊትን **ንጹህ** ማድረግ እና የአካባቢን **ንጽህና** ማሻሻልን ለመተግበር የሚረዳው የተቀናጀ የውሃ አቅርቦት የግል እና የአካባቢ ንፅህና አጠባበቅ (WASH) ኢንተርቬንሺን ከፍተኛ የትራኮማ ሥርጭት ባለበት እና የትራኮማ መድሃኒት ሥርጭት በተቋረጠበት አካባቢ የአኩላር ክላምዲያ ሥርጭት መጨመርን መከላከል አልቻለም። ለጥናቱ በተመረጡት 40 ክላስተሮች ላይ በቀጣይነት የትራኮማ መድሃኒት ሥርጭት እየተካሄደ ባለበት ሁኔታ የ WASH ኢንተርቬንሺን በትራኮማ ሥርጭት ላይ የሚያስገኘውን ውጤት ና የሚያስከትለውን ለውጥ ለማጥናት ይህ ጥናት በቀጣይነት እየተካሄደ ይገኛል። ያልተቋረጠ ከፍተኛ የትራኮማ ሥርጭት ባለባቸው አካባቢዎች የትራኮማ መድሃኒት ሥርጭት ሳይቋረጥ የመተግበር አሥፈላጊነትን ያመለክታል።

የገንዘብ ድጋፍ ምንጭ ፡ ናሽናል ኢኒስቲቲዩት ኦፍ ሔልዝ - ናሽናል አይ ኢኒስቲቲዩት
