# Supplementary material for: Water, sanitation, and hygiene for control of trachoma in Ethiopia (WUHA): a two-arm, parallel-group, cluster-randomised trial
Source: Lancet Glob Health. Author manuscript; Available in PMC 2022 Aug 9. (PMC9360557; doi:10.1016/S2214-109X(21)00409-5)
Supplement: 2 [file NIHMS1827832-supplement-2.pdf]

THE LANCET
Global Health

Supplementary appendix 2

This appendix formed part of the original submission and has been peer reviewed. We post it as supplied by the authors.

Supplement to: Aragie S, Wittberg DM, Tadesse W, et al. Water, sanitation, and hygiene for control of trachoma in Ethiopia (WUHA): a two-arm, parallel-group, cluster-randomised trial. *Lancet Glob Health* 2022; **10:** e87–95.

ONLINE MATERIAL

Table of Contents

Supplementary Table 1. Prevalence of ocular chlamydia at annual study visits, ages 0-5 years..…………................................... 2
Supplementary Table 2. Prevalence of ocular chlamydia at annual study visits, ages 6-9 years..…………................................... 3
Supplementary Table 3. Prevalence of ocular chlamydia at annual study visits, ages ≥10 years..……...….................................. 4
Supplementary Table 4. Chlamydial load index at annual study visits, ages 0-5 years..…………………………………………….. 5
Supplementary Table 5. Seropositivity at baseline and month 36 in children aged 1-2 years…………………………………….. 6
Supplementary Table 6. Cluster-randomized trials assessing face hygiene or environmental improvements for trachoma………. 7

Supplementary Table 1. Prevalence of ocular chlamydia at annual study visits, ages 0-5 years. Numerators indicate the number of individuals per cluster testing positive and denominators the total number of randomly selected individuals per cluster in which a conjunctival swab was collected.

	Number positive out of total tested per cluster (%)			
**Cluster**	**Month 0**	**Month 12**	**Month 24**	**Month 36**
**WASH**				
1	6/34 (18%)	14/29 (48%)	12/34 (35%)	12/41 (29%)
2	5/23 (22%)	12/29 (41%)	10/29 (34%)	14/28 (50%)
3	4/30 (13%)	10/29 (34%)	12/35 (34%)	4/34 (12%)
4	1/27 (4%)	12/30 (40%)	11/33 (33%)	7/33 (21%)
5	8/35 (23%)	5/34 (15%)	4/34 (12%)	3/28 (11%)
6	5/31 (16%)	2/29 (7%)	17/36 (47%)	14/32 (44%)
7	1/33 (3%)	4/33 (12%)	12/33 (36%)	20/32 (63%)
8	5/36 (14%)	11/28 (39%)	18/36 (50%)	20/34 (59%)
9	1/34 (3%)	2/35 (6%)	3/34 (9%)	16/35 (46%)
10	7/36 (19%)	7/36 (19%)	10/31 (32%)	13/33 (39%)
11	2/30 (7%)	8/30 (27%)	11/36 (31%)	11/35 (31%)
12	1/30 (3%)	0/32 (0%)	1/30 (3%)	4/34 (12%)
13	0/32 (0%)	1/30 (3%)	2/32 (6%)	1/33 (3%)
14	2/35 (6%)	2/32 (6%)	5/31 (16%)	11/30 (37%)
15	5/29 (17%)	7/29 (24%)	6/21 (29%)	3/18 (17%)
16	2/36 (6%)	25/34 (74%)	21/34 (62%)	17/32 (53%)
17	10/24 (42%)	12/20 (60%)	9/14 (64%)	6/10 (60%)
18	1/34 (3%)	2/35 (6%)	0/37 (0%)	5/34 (15%)
19	1/24 (4%)	0/15 (0%)	3/25 (12%)	6/21 (29%)
20	0/19 (0%)	0/17 (0%)	3/19 (16%)	5/29 (17%)
**Control**				
21	0/36 (0%)	2/30 (7%)	1/36 (3%)	17/34 (50%)
22	1/33 (3%)	8/27 (30%)	12/29 (41%)	20/31 (65%)
23	0/35 (0%)	6/32 (19%)	10/36 (28%)	12/33 (36%)
24	11/32 (34%)	6/21 (29%)	11/22 (50%)	15/20 (75%)
25	10/33 (30%)	7/30 (23%)	11/35 (31%)	10/34 (29%)
26	9/31 (29%)	4/25 (16%)	8/26 (31%)	8/32 (25%)
27	7/33 (21%)	7/30 (23%)	7/26 (27%)	9/29 (31%)
28	8/38 (21%)	8/25 (32%)	4/36 (11%)	9/40 (23%)
29	1/32 (3%)	7/31 (23%)	12/35 (34%)	20/31 (65%)
30	1/36 (3%)	3/31 (10%)	2/33 (6%)	8/31 (26%)
31	0/32 (0%)	4/31 (13%)	11/36 (31%)	9/36 (25%)
32	0/38 (0%)	2/32 (6%)	0/31 (0%)	0/35 (0%)
33	1/36 (3%)	1/32 (3%)	3/27 (11%)	11/31 (35%)
34	4/34 (12%)	11/32 (34%)	11/29 (38%)	17/31 (55%)
35	4/33 (12%)	6/29 (21%)	8/35 (23%)	10/34 (29%)
36	13/30 (43%)	9/34 (26%)	5/28 (18%)	2/20 (10%)
37	1/24 (4%)	5/29 (17%)	5/23 (22%)	4/21 (19%)
38	0/38 (0%)	0/22 (0%)	0/32 (0%)	0/20 (0%)
39	0/24 (0%)	9/28 (32%)	7/32 (22%)	3/22 (14%)
40	1/18 (6%)	3/17 (18%)	2/9 (22%)	1/12 (8%)

Supplementary Table 2. Prevalence of ocular chlamydia at annual study visits, ages 6-9 years. Numerators indicate the number of individuals per cluster testing positive and denominators the total number of randomly selected individuals per cluster in which a conjunctival swab was collected. At month 0 the number testing positive was estimated with maximum likelihood methods from pooled swab results.

	Number positive out of total tested per cluster (%)			
**Cluster**	**Month 0**	**Month 12**	**Month 24**	**Month 36**
**WASH**				
1	5/36 (14%)	9/30 (30%)	14/38 (37%)	15/34 (44%)
2	2/24 (8%)	11/30 (37%)	9/24 (38%)	7/17 (41%)
3	2/33 (6%)	3/30 (10%)	11/36 (31%)	6/34 (18%)
4	1/28 (4%)	4/27 (15%)	5/34 (15%)	7/29 (24%)
5	6/24 (25%)	8/28 (29%)	6/32 (19%)	5/33 (15%)
6	3/30 (10%)	3/34 (9%)	13/38 (34%)	11/36 (31%)
7	0/34 (0%)	1/29 (3%)	13/33 (39%)	15/34 (44%)
8	0/35 (0%)	7/33 (21%)	16/33 (48%)	13/35 (37%)
9	1/29 (3%)	4/34 (12%)	8/36 (22%)	12/36 (33%)
10	5/36 (14%)	9/34 (26%)	10/37 (27%)	12/33 (36%)
11	4/24 (17%)	3/29 (10%)	10/35 (29%)	17/34 (50%)
12	0/32 (0%)	1/29 (3%)	1/33 (3%)	4/33 (12%)
13	1/29 (3%)	1/30 (3%)	2/35 (6%)	2/33 (6%)
14	0/33 (0%)	0/29 (0%)	1/22 (5%)	5/25 (20%)
15	0/20 (0%)	2/22 (9%)	4/22 (18%)	4/24 (17%)
16	0/18 (0%)	10/21 (48%)	18/27 (67%)	20/41 (49%)
17	5/13 (38%)	5/12 (42%)	10/17 (59%)	7/15 (47%)
18	0/19 (0%)	0/31 (0%)	1/36 (3%)	5/35 (14%)
19	0/17 (0%)	3/19 (16%)	2/32 (6%)	5/24 (21%)
20	1/15 (7%)	0/11 (0%)	0/16 (0%)	1/15 (7%)
**Control**				
21	5/33 (15%)	2/31 (6%)	6/34 (18%)	16/34 (47%)
22	2/30 (7%)	1/30 (3%)	12/35 (34%)	13/31 (42%)
23	0/35 (0%)	2/31 (6%)	4/34 (12%)	11/35 (31%)
24	7/34 (21%)	4/27 (15%)	14/32 (44%)	16/28 (57%)
25	7/30 (23%)	5/33 (15%)	8/33 (24%)	7/35 (20%)
26	5/30 (17%)	2/23 (9%)	9/25 (36%)	10/33 (30%)
27	10/36 (28%)	4/30 (13%)	7/34 (21%)	5/33 (15%)
28	5/35 (14%)	3/30 (10%)	10/33 (30%)	5/33 (15%)
29	1/33 (3%)	4/29 (14%)	5/37 (14%)	24/33 (73%)
30	1/36 (3%)	4/33 (12%)	6/28 (21%)	13/36 (36%)
31	0/33 (0%)	2/28 (7%)	4/37 (11%)	7/33 (21%)
32	0/40 (0%)	1/30 (3%)	0/20 (0%)	0/37 (0%)
33	1/30 (3%)	3/26 (12%)	1/33 (3%)	5/31 (16%)
34	3/37 (8%)	7/36 (19%)	12/34 (35%)	12/36 (33%)
35	1/34 (3%)	2/28 (7%)	5/31 (16%)	6/34 (18%)
36	3/24 (13%)	6/22 (27%)	6/21 (29%)	2/22 (9%)
37	0/17 (0%)	4/24 (17%)	2/15 (13%)	4/20 (20%)
38	0/22 (0%)	0/25 (0%)	0/37 (0%)	0/25 (0%)
39	1/17 (6%)	5/19 (26%)	5/26 (19%)	6/27 (22%)
40	0/14 (0%)	0/13 (0%)	0/11 (0%)	1/16 (6%)

Supplementary Table 3. Prevalence of ocular chlamydia at annual study visits, ages ≥ 10 years. Numerators indicate maximum likelihood estimates of the number of individuals per cluster testing positive, assessed from pooled conjunctival swabs. Denominators indicate the total number of randomly selected individuals per cluster in which a conjunctival swab was collected. No swabs were collected for this age group at months 12 or 24.

	Estimated number positive out of total tested per cluster (%)	
Cluster	Month 0	Month 36
**WASH**		
1	1/28 (4%)	3/28 (11%)
2	1/25 (4%)	0/14 (0%)
3	0/31 (0%)	1/33 (3%)
4	0/31 (0%)	3/31 (10%)
5	1/35 (3%)	2/35 (6%)
6	2/31 (6%)	5/31 (16%)
7	1/27 (4%)	2/37 (5%)
8	0/29 (0%)	3/33 (9%)
9	0/28 (0%)	0/33 (0%)
10	1/33 (3%)	2/32 (6%)
11	1/22 (5%)	1/30 (3%)
12	2/28 (7%)	0/28 (0%)
13	0/28 (0%)	0/30 (0%)
14	0/31 (0%)	0/29 (0%)
15	1/30 (3%)	1/32 (3%)
16	2/27 (7%)	10/28 (36%)
17	5/31 (16%)	3/30 (10%)
18	0/28 (0%)	0/33 (0%)
19	1/32 (3%)	1/29 (3%)
20	0/33 (0%)	0/30 (0%)
**Control**		
21	0/27 (0%)	1/31 (3%)
22	0/21 (0%)	1/34 (3%)
23	0/28 (0%)	0/28 (0%)
24	0/31 (0%)	3/25 (12%)
25	1/24 (4%)	1/32 (3%)
26	1/30 (3%)	1/31 (3%)
27	0/29 (0%)	0/27 (0%)
28	0/31 (0%)	3/33 (9%)
29	1/27 (4%)	3/25 (12%)
30	0/29 (0%)	1/29 (3%)
31	0/28 (0%)	1/28 (4%)
32	0/35 (0%)	0/32 (0%)
33	1/33 (3%)	0/31 (0%)
34	1/31 (3%)	0/32 (0%)
35	0/27 (0%)	3/32 (9%)
36	1/31 (3%)	1/31 (3%)
37	0/28 (0%)	1/33 (3%)
38	0/25 (0%)	0/16 (0%)
39	0/33 (0%)	1/30 (3%)
40	0/32 (0%)	1/32 (3%)

Supplementary Table 4. Chlamydial load index at annual study visits, ages 0-5 years. Values indicate the cluster-level mean of the log-transformed elementary body counts, assessed among those children in the 0-5 year-old cross-sectional random sample with a positive polymerase chain reaction (PCR) test for chlamydia. PCR was done on the m2000 RealTi*me* PCR platform (Abbott Molecular Inc., DesPlaines IL). Elementary body counts were standardized against quantified elementary body suspensions provided by the University of California, San Francisco.

	Number of positive PCR tests and chlamydial load index per community							
	Month 0		Month 12		Month 24		Month 36	
Cluster	Number positive	Mean load	Number positive	Mean load	Number positive	Mean load	Number positive	Mean load
**WASH**								
1	6	5.3	14	6.8	12	5.0	12	3.2
2	5	7.4	12	4.6	10	5.7	14	4.3
3	4	5.2	10	2.7	12	4.6	4	4.3
4	1	10.3	12	9.2	11	4.6	7	3.6
5	8	5.5	5	1.5	4	3.9	3	3.5
6	5	3.5	2	7.8	17	6.4	14	4.2
7	1	3.1	4	11.8	12	6.9	20	6.7
8	5	3.5	11	7.0	18	6.6	20	3.8
9	1	1.5	2	12.4	3	4.7	16	5.2
10	7	4.2	7	5.2	10	5.2	13	3.9
11	2	1.6	8	5.4	11	4.4	11	5.6
12	1	1.8	0	---	1	2.8	4	2.7
13	0	---	1	-2.1	2	5.3	1	3.4
14	2	2.0	2	3.3	5	4.9	11	5.0
15	5	7.1	7	3.7	6	4.9	3	4.3
16	2	4.5	25	6.0	21	6.0	17	3.2
17	10	4.7	12	5.9	9	4.4	6	2.5
18	1	2.3	2	2.6	0	---	5	5.5
19	1	7.3	0	---	3	7.1	6	4.5
20	0	---	0	---	3	3.1	5	4.4
**Control**								
21	0	---	2	6.2	1	6.0	17	6.4
22	1	4.6	8	5.7	12	6.1	20	5.0
23	0	---	6	5.8	10	4.5	12	5.1
24	11	0.9	6	3.8	11	5.7	15	3.2
25	10	0.6	7	7.6	11	6.4	10	3.4
26	9	-0.3	4	3.7	8	4.6	8	2.0
27	7	4.0	7	6.1	7	5.6	9	2.9
28	8	5.6	8	4.3	4	5.6	9	3.2
29	1	1.0	7	6.3	12	6.3	20	4.9
30	1	1.1	3	5.7	2	8.5	8	6.2
31	0	---	4	9.1	11	7.5	9	3.6
32	0	---	2	5.9	0	---	0	---
33	1	1.3	1	7.4	3	7.1	11	4.1
34	4	2.9	11	6.6	11	5.8	17	4.2
35	4	4.0	6	3.6	8	4.9	10	3.5
36	13	2.3	9	3.2	5	4.2	2	2.8
37	1	4.0	5	1.5	5	3.9	4	4.1
38	0	---	0	---	0	---	0	---
39	0	---	9	4.6	7	4.5	3	4.6
40	1	0.6	3	4.2	2	2.2	1	8.6

Supplementary Table 5. Seropositivity at baseline and month 36 in children aged 1-2 years. Values indicate the cluster-level proportion of children aged 12 months up to but not including 36 months whose dried blood spots tested positive for both Pgp3 and CT694 antibodies. A random sample of children was chosen at each time point. Age was reported by caregivers at a census performed one month prior. Serologic testing was done at the Centers for Disease Control and Prevention (Atlanta, Georgia) on the Luminex platform.

	Estimated number positive out of total tested per cluster (%)	
**Cluster**	**Month 0**	**Month 36**
**WASH**		
1	2/7 (29%)	6/14 (43%)
2	6/14 (43%)	5/13 (38%)
3	2/11 (18%)	0/14 (0%)
4	2/14 (14%)	2/13 (15%)
5	2/14 (14%)	1/5 (20%)
6	3/13 (23%)	2/14 (14%)
7	2/12 (17%)	7/10 (70%)
8	3/12 (25%)	4/11 (36%)
9	1/8 (13%)	6/18 (33%)
10	6/11 (55%)	4/11 (36%)
11	2/6 (33%)	3/10 (30%)
12	0/10 (0%)	2/10 (20%)
13	0/11 (0%)	1/13 (8%)
14	0/9 (0%)	1/8 (13%)
15	3/13 (23%)	1/2 (50%)
16	3/13 (23%)	6/11 (55%)
17	6/8 (75%)	1/1 (100%)
18	0/9 (0%)	2/12 (17%)
19	0/7 (0%)	4/7 (57%)
20	0/7 (0%)	0/7 (0%)
**Control**		
21	3/13 (23%)	2/9 (22%)
22	1/9 (11%)	5/7 (71%)
23	1/10 (10%)	2/9 (22%)
24	3/14 (21%)	7/7 (100%)
25	5/11 (45%)	5/11 (45%)
26	4/8 (50%)	3/11 (27%)
27	3/14 (21%)	3/10 (30%)
28	3/12 (25%)	5/16 (31%)
29	1/10 (10%)	3/8 (38%)
30	2/8 (25%)	0/8 (0%)
31	1/11 (9%)	3/9 (33%)
32	0/10 (0%)	1/8 (13%)
33	1/13 (8%)	2/7 (29%)
34	3/11 (27%)	4/11 (36%)
35	1/12 (8%)	2/9 (22%)
36	1/9 (11%)	1/6 (17%)
37	0/9 (0%)	2/6 (33%)
38	0/8 (0%)	0/8 (0%)
39	1/8 (13%)	0/5 (0%)
40	3/8 (38%)	1/2 (50%)

Supplementary Table 6. Cluster-randomized trials assessing face hygiene or environmental improvements for trachoma.

Study	Clusters	Comparison Groups[a]	Outcome[b]	Finding
Peach (1987)[1]	N=9<br>N=9<br>N=9<br>N=9	1) Antibiotics<br>2) Face washing<br>3) Face washing, antibiotics<br>4) No intervention	TF at 3 m	No difference
Resnikoff (1995)[2]	N=1<br>N=1<br>N=1<br>N=1	1) Hygiene education<br>2) Antibiotics<br>3) Hygiene education, antibiotics<br>4) No intervention	TF/TI at 6 m	Less TF/TI in education group compared with no intervention[c]
West (1995)[3]	N=3<br>N=3	1) Face-washing education, antibiotics<br>2) Antibiotics only	TF, TI at 1 y	No difference in TF; less TI in face-washing group[d]
Emerson (2004)[4]	N=7<br>N=7<br>N=7	1) Latrines<br>2) Insecticide<br>3) No intervention	TF/TI at 6 m	Less TF/TI in insecticide group, but not in latrine group[e]
Atik (2006)[5]	N=1<br>N=1<br>N=1	1) Surgery, targeted antibiotics<br>2) Surgery, targeted antibiotics, F&E<br>3) Surgery only	CT at 3 y	No difference
Edwards (2006)[6]	N=10<br>N=20	1) Video education, NGO activities<br>2) NGO activities only	TF/TI at 1 y	No difference
West (2006)[7]	N=8<br>N=8	1) Insecticide, antibiotics<br>2) Antibiotics only	TF/TI/CT at 6 m	No difference
Abdou (2010)[8]	N=5<br>N=5	1) Wells, health education, antibiotics<br>2) Antibiotics only	CT at 2 y	No difference
Stoller (2011)[9]	N=12<br>N=12	1) Latrines, antibiotics<br>2) Antibiotics only	CT at 2 y	No difference
Aragie (2021)[10]	N=7<br>N=7	1) Hand dug wells<br>2) No intervention	CT at 2y	No difference

[a] Antibiotics=mass antibiotic distribution with either a single round of oral azithromycin (Abdou, Stoller, West 2006), a 1-month course of topical tetracycline (West 1995), a 1-week course of topical tetracycline each month for 3 months (Peach), or antibiotics targeted to households of children aged 5-15 y with TF/TI (Atik); F&E=facial cleanliness and environmental improvement (details of intervention not provided); NGO=non-governmental organization

[b] CT=*Chlamydia trachomatis* from conjunctival swab, assessed with polymerase chain reaction; TF=trachomatous inflammation—follicular; TI= trachomatous inflammation—intense; PCR=polymerase chain reaction evidence of ocular chlamydia infection

[c] OR 0.42 (95%CI 0.20-0.91) in analysis not accounting for cluster randomization

[d] OR 0.62 (95%CI 0.40-0.97) in analysis not accounting for cluster randomization

[e] 7.2 fewer cases of trachoma per 100,000 in insecticide villages; P=0.005

References:

1. Peach H, Piper S, Devanesen D, et al. Northern Territory trachoma control and eye health committee's randomised controlled trial of the effect of eye drops and eye washing on follicular trachoma among Aboriginal children, 1987.
2. Resnikoff S, Peyramaure F, Bagayogo CO, Huguet P. Health education and antibiotic therapy in trachoma control. *Rev Int Trach Pathol Ocul Trop Subtrop Sante Publique* 1995; **72**: 89-98, 101-10.
3. West S, Munoz B, Lynch M, et al. Impact of face-washing on trachoma in Kongwa, Tanzania. *Lancet* 1995; **345**(8943): 155-8.
4. Emerson PM, Lindsay SW, Alexander N, et al. Role of flies and provision of latrines in trachoma control: cluster-randomised controlled trial. *Lancet* 2004; **363**(9415): 1093-8.
5. Atik B, Thanh TT, Luong VQ, Lagree S, Dean D. Impact of annual targeted treatment on infectious trachoma and susceptibility to reinfection. *JAMA* 2006; **296**(12): 1488-97.
6. Edwards T, Cumberland P, Hailu G, Todd J. Impact of health education on active trachoma in hyperendemic rural communities in Ethiopia. *Ophthalmology* 2006; **113**(4): 548-55.
7. West SK, Emerson PM, Mkocha H, et al. Intensive insecticide spraying for fly control after mass antibiotic treatment for trachoma in a hyperendemic setting: a randomised trial. *Lancet* 2006; **368**(9535): 596-600.
8. Abdou A, Munoz BE, Nassirou B, et al. How much is not enough? A community randomized trial of a Water and Health Education programme for Trachoma and Ocular C. trachomatis infection in Niger. *Trop Med Int Health* 2010; **15**(1): 98-104.
9. Stoller NE, Gebre T, Ayele B, et al. Efficacy of latrine promotion on emergence of infection with ocular Chlamydia trachomatis after mass antibiotic treatment: a cluster-randomized trial. *Int Health* 2011; **3**(2): 75-84.
10. Aragie S, Gebresillasie S, Chernet A, et al. Community Hand-Dug Wells for Trachoma: A Cluster-Randomized Trial. *Am J Trop Med Hyg* 2021; **104**(4):1271-7.
